# Cervical ripening with vaginal Misoprostol plus Hyoscine-N-Butylbromide versus vaginal Misoprostol alone among pregnant women: a double-blind randomised controlled trial

**DOI:** 10.4314/gmj.v58i1.7

**Published:** 2024-03

**Authors:** Ifeakachukwu D Agadaga, Peter N Ebeigbe, Babatunde Oyefara

**Affiliations:** 1 Department of Obstetrics and Gynaecology, Federal Medical Centre, Asaba, Delta State, Nigeria; 2 Department of Obstetrics and Gynaecology, Delta State University, Abraka, Delta State, Nigeria

**Keywords:** Cervical ripening, Induction of labour, Bishop's score, Misoprostol, Hyoscine

## Abstract

**Objective:**

To compare cervical ripening time with the use of vaginal Misoprostol plus Hyoscine-N-Butylbromide, with vaginal Misoprostol alone.

**Design:**

A double-blind randomized controlled trial with Pan-African Clinical Trials Registry (PACTR) approval number PACTR202112821475292

**Setting:**

Federal Medical Centre, Asaba, Nigeria.

**Participants:**

A total of 126 eligible antenatal patients for cervical ripening were enrolled.

**Interventions:**

Participants in Group A had 25µg of vaginal misoprostol with 1ml of intramuscular placebo, and those in Group B had 25µg of vaginal misoprostol with 20mg of Intramuscular Hyoscine (1 ml). Oxytocin infusion was used when indicated, and the labour was supervised as per departmental protocol.

**Main outcome measure:**

Cervical ripening time

**Results:**

The mean cervical ripening time was statistically significantly shorter in the hyoscine group (8.48±4.36 hours) than in the placebo group (11.40±7.33 hours); p-value 0.02, 95% CI 0.80-5.05. There was no statistically significant difference in the mean induction-delivery interval in Group A (7.38±5.28 hours) compared to Group B (7.75±5.04 hours), with a value of 0.54. The mode of delivery was comparable. However, women in Group B (53, 84.1%) achieved more vaginal deliveries than women in Group A (50, 79.4%); p-value 0.49. Thirteen women in Group A (20.6%) had a caesarean section, while ten women (15.9%) in Group B had a caesarean section (p-value 0.49, RR 0.94, CI 0.80-1.11). Adverse maternal and neonatal outcomes were not statistically significant between the two groups.

**Conclusion:**

Intramuscular hyoscine was effective in reducing cervical ripening time when used as an adjunct to vaginal Misoprostol, with no significant adverse maternal or neonatal outcome.

**Funding:**

None declared

## Introduction

The status of the cervix has an impact on the duration of induction of labour and the likelihood of vaginal delivery.^1^ Thus, it is pertinent to ascertain the favourability of the cervix before induction of labour. Cervical ripening refers to the softening and distensibility of the cervix in preparation for labour and delivery.^2^ It is usually employed when the cervix is unfavourable.

Induction of labour is defined as the artificial initiation of labour, after the age of viability, in a parturient with an intact fetal membrane with the aim of achieving vaginal delivery.^3^ It is usually indicated in conditions where the benefits of delivery of the fetus outweigh the risk of continuing the pregnancy, like prolonged pregnancy, hypertensive disorder and diabetes in pregnancy. 4 There are several agents for cervical ripening, ranging from pharmacological to mechanical methods. Misoprostol is a prostaglandin E1 analogue (PGE1) which causes contraction of uterine smooth muscles, and pharmacokinetic studies carried out during pregnancy showed that it is effectively absorbed across the vagina mucosa.^5^ There are risks associated with the use of vaginal Misoprostol alone, which include tachysystole, hypertonus, uterine rupture, fetal distress and a higher intervention rate. These are related to the dose and dosing interval of the misoprostol.^6^ They are commoner when a higher dose is used. Though the lower dose, like 25µg, is associated with fewer complications, it requires more oxytocin augmentation with its attendant risk and increased rate of intervention. It also requires more frequent dosing which translates to multiple vaginal examinations.^6^, ^7^

Hyoscine -N- butyl bromide (HBB) is a quaternary ammonium compound obtained from the Dubosia tree found mainly in Australia^8^ and a peripherally acting, competitive antagonist of acetylcholine at the muscarinic receptors. It selectively acts on the intramural parasympathetic ganglia, especially the cervico-uterine plexus, aiding analgesia and cervical dilatation without affecting uterine contractions. It has been shown to reduce the duration of labour by overcoming cervical spasms and promoting cervical dilatation with no adverse fetal outcome.^8^, ^9^, ^10^

Based on the findings of the positive effects of HBB in facilitating the first stage of labour, there is a strong possibility that it may have similar effects on cervical ripening and thus ameliorate the aforementioned shortcomings of vaginal misoprostol when used alone.

There is very limited information on the likely effects of the use of HBB as an adjunct to vaginal misoprostol for cervical ripening. Due to the fact that it may reduce the time needed to achieve cervical ripening as well as the side effects of vaginal misoprostol, there is a need for a study to explore this theme.

The aim of the study was to compare the duration of cervical ripening time with the use of vaginal misoprostol plus Hyoscine-N-Butylbromide with vaginal misoprostol alone, among antenatal women in Federal Medical Centre, Asaba, Nigeria.The specific objectives were to determine the cervical ripening time, induction-delivery interval, rate of vaginal delivery, rate of operative delivery, rate of adverse maternal and fetal outcomes in women who had 25µg vaginal misoprostol alone, and in those who had 20mg intramuscular Hyoscine-N-Butylbromide plus 25µg vaginal misoprostol for cervical ripening; and to make recommendations on the best option of the two approaches studied for cervical ripening based on the comparison of the findings of the previous objectives.

## Methods

### Study design

This was a double-blind randomized controlled trial of women who met the inclusion criteria at Federal Medical Centre, Asaba.

### Study area

The study was conducted in the Department of Obstetrics and Gynaecology, Federal Medical Centre, Asaba, Delta state, south-south, Nigeria, between December 15^th^, 2021 and October 20^th^, 2022. The hospital is a tertiary healthcare institution that serves as a referral centre for primary and secondary healthcare facilities located in Delta state and other neighbouring states like Edo, Anambra and Kogi.

### Study population

These were all consenting antenatal patients seen in the antenatal clinic and ward for cervical ripening and induction of labour during the period of the study and who met the inclusion criteria until the calculated sample size was reached.

### Inclusion criteria

All consenting participants, singleton pregnancy, cephalic presentation, intact fetal membrane, gestational age ≥ 37 weeks, absence of spontaneous uterine contraction, Bishop score ≤ 5 and a reactive non stress test.

### Exclusion criteria

Participant's refusal, multiple pregnancy, abnormal fetal presentation, ruptured fetal membrane, gestational age < 37 weeks, presence of spontaneous uterine contraction, fetal congenital abnormality, intrauterine fetal death, previous uterine surgery, grandmultiparity, abnormal nonstress test, Bishop score ≥ 6, contraindication to vaginal delivery, any known allergy to misoprostol and/or hyoscine.

### Sample size determination

A sample size of 126 participants (63 participants in each group), with provision for 10% attrition, was calculated to achieve a power of 80% and detect a significance of less than 5%.

The sample size was calculated with the formula^11^;


$$N=\frac{(\sigma_{1}^{2}+\sigma_{2}^{2})\times (Z_{1-\alpha /2}+Z_{1-\beta }{)}^{2}}{(M_{1}-M_{2}{)}^{2}}$$

Where: N = minimum sample size required in each study group, σ_1_= standard deviation of the outcome variable in group 1, σ2= standard deviation of the outcome variable in group 2, Z1_−α/2_ = standard normal deviate at 5% significance (Z table); 1.96, Z_1−β_ = standard normal deviate for power of 80% (Z table); 0.842, M_1_ = mean of the outcome variable in group 1 and M2= mean of the outcome variable in group 2.

The means and standard deviations of the outcome variables were based on a study by Aduloju et al^12^, who found that combining different agents for cervical ripening was statistically significantly effective in reducing the duration of cervical ripening with no adverse maternal or fetal outcome when compared to a single agent^12^.

They compared combined Foley's catheter and vaginal misoprostol to Foley's catheter or low-dose vaginal misoprostol alone on cervical ripening time. The mean cervical ripening time (hours) was 9.30 ± 2.56 and 8.08 ± 2.06 in the misoprostol alone and combined misoprostol and catheter groups respectively^12^. This reported cervical ripening time was used for the sample size calculation.

### Randomization, allocation sequence and blinding

Participants admitted for cervical ripening and induction of labour who met the inclusion criteria and consented were recruited using a computer-generated randomization schedule (Stat Trek's Random Number Generator).^13^ The women were assigned these computed generated random numbers until the 126 numbers (sample size) were exhausted.

An independent pharmacist was involved in the preparation of the Hyoscine and the placebo (water for injection). Under an aseptic condition, 504 syringes (126 x 4) were prepared in batches and stored in the fridge. Two hundred and fifty-two (252) syringes contained 20mg of HBB (1 ml), and the other 252 contained 1 ml of water for injection. Both HBB and water for injection (placebo) were colourless, so the syringes containing the active drug and placebo were indistinguishable. The pharmacist randomly assigned numbers to the syringes in groups of 4 and was the only person who knew the content of the syringes. The random number assigned to the woman was matched with the corresponding random number on the prepared syringes that were administered. The study participants and outcome measure assessors, including the researchers, were blinded to the group allocation of participants. At the end of the study, the pharmacist decoded the random numbers of the syringes and their contents for the researchers and divided them into two groups before data analysis.

### Study procedure

A preliminary admission biophysical profile was done to assess the fetal status, in addition to other basic investigations (packed cell volume, urinalysis, grouping and cross-matching of blood). The vaginal examination was done for the modified Bishop Score assessment, and vaginal Misoprostol was administered (25µg six hourly), while the attending nurse administered the intramuscular (IM) drug, which was either Hyoscine or placebo, 1ml every 6 hours.

### Group A (combined misoprostol and placebo)

The participants in this group received intermittent 25µg Misoprostol, inserted into the posterior vaginal fornix every 6 hours until the cervix was favourable (Bishop's score ≥ 6) to a maximum of 4 doses (departmental protocol), with a concurrent IM administration of 1 ml of placebo. The cervix was assessed and scored at each examination. They were reviewed before the stipulated time if they were contracting.

The administration of the drugs was discontinued when a favourable cervix was diagnosed.

### Group B (combined Hyoscine and misoprostol)

Participants assigned to this group received vaginal misoprostol as described above with concurrent IM administration of 1 ml of Hyoscine, and cervical status scored at each examination.

### Oxytocin administration and labour management

In both groups, oxytocin infusion was commenced in women with favourable Bishop Score (≥ 6) and in those that had not developed active phase labour. It was delayed for 6 hours after the last dose of vaginal misoprostol and commenced after the artificial rupture of the membrane. The departmental protocol of oxytocin administration was adhered to by adding 5IU (International unit) of oxytocin into 500ml of normal saline, starting with 5mIU per minute (10 drops per minute) and increasing every 30 minutes to achieve 3 adequate uterine contractions in 10 mins, or to a maximum of 30mIU per minute (60 drops per minute).

Following the diagnosis of active phase labour, routine active management of labour was adopted regardless of the study group, using a partograph. The patients were on continuous cardiotocographic monitoring. The pulse rate, fetal heart rate and uterine contraction were monitored every 30 minutes, while the blood pressure and temperature were monitored every 4 hours. A vaginal examination was done every 4 hours except when indicated to assess the progress of labour, and the colour of amniotic fluid was recorded at each examination. Analgesia in labour was with the use of intramuscular pentazocin and promethazine but withheld if delivery was anticipated within 4 hours to avoid neonatal respiratory depression.

In the second stage of labour, the fetal heart rate was checked every 5 minutes, the patients were encouraged to bear down with each uterine contraction, the perineum guarded, and episiotomy was given in those with rigid perineum and threatened perineal tear. The baby was delivered and handed over to the attending neonatologist for possible resuscitation. Uterine hyperstimulation and fetal heart irregularities recorded were managed by discontinuing any oxytocin infusion, intrauterine resuscitation and emergency delivery (via the most expedient route) if unresponsive to the resuscitation. The participants were educated on the possible side effects of the drugs, and any noted adverse drug reaction was managed accordingly. Their vital signs were monitored regularly.

### Outcome measures

The primary outcome measure was cervical ripening time, while the secondary outcome measures were induction-delivery interval, oxytocin requirement, rate of vaginal and operative deliveries, Apgar scores at 1 and 5 minutes, Admission into the neonatal unit (NNU), complications from cervical ripening (uterine tachysystole, uterine hypertonus, hyperstimulation, fetal heart abnormalities, uterine rupture), side effects of Hyoscine (blurred vision, tachycardia, dry mouth, hypersensitivity reaction, urinary retention) and Misoprostol (fever, nausea, vomiting, diarrhoea).

### Data analysis

The data collected (using a predesigned proforma) was analyzed using IBM SPSS (Statistical Product and Service Solutions) version 26 for Windows.^14^ Data analysis was by intention-to-treat to ensure unbiased comparison. The outcome measures were compared between the study groups. Categorical data were expressed as absolute numbers and percentages, while the continuous variables were analyzed using mean ± standard deviation. Qualitative variables were compared between the two groups using the χ^2^ (chi-square) test and the Fisher exact test where appropriate, while the quantitative variables were compared by an independent sample t-test for parametric data and by the Mann-Whitney U test for non-parametric data. The 95 % confidence interval was used, the level of statistical significance was set at a P-value of < 0.05 and relative risk (RR) was used where applicable.

### Ethical consideration

Approval to carry out this study was sought and obtained from the Research and Ethics committee of Federal Medical Centre, Asaba, in conformity with the Helsinki Declaration.15 The ethical approval number is FMC/ASB/A81 VOL.XIII/196. The study protocol was registered with the Pan African Clinical Trial Registry (PACTR), with approval number PACTR202112821475292.

## Results

**Figure 1:**
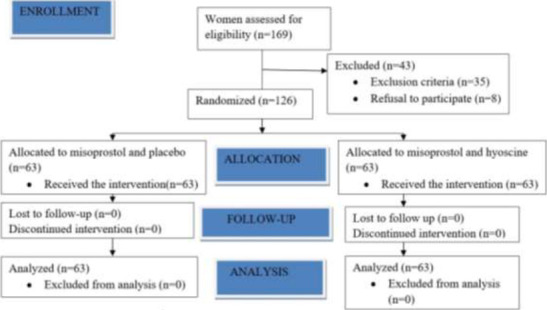
Study flow chart

A total of 169 women were assessed for eligibility for the study, but 126 were enrolled during the study period; 35 women did not meet the inclusion criteria, while 8 potential participants did not consent to the study. All the women enrolled participated to the end. Sixty-three (63) participants in Group A received 25µg vaginal Misoprostol and intramuscular placebo (1ml of water for injection), while the remaining 63 participants in Group B were administered 25µg vaginal Misoprostol and 20mg intramuscular Hyoscine (1ml). The study flow chart is shown in Figure 1.

Table 1 shows the demographic characteristics of the study participants and the indications for cervical ripening. There were no significant differences between the two groups.

**Table 1:** Demographic characteristics of participants and indications of cervical ripening

	Mode of cervical ripening	Mode of cervical ripening			
	Group A; Misoprostol + Placebo (n = 63)	Group B; Misoprostol + Hyoscine (n = 63)	Test statistics	*p-value*	95% Confidence interval (CI)
**Variables**					
**Age(years)**	30.25±5.01	29.75± 5.26	0.55^t^	0.58	−1.30-2.32
**Parity**	0.86±1.16	0.67±1.03	1833.00^u^	0.43	−0.20-0.58
**Gestational age (weeks)**	39.83±1.62	40.02±1.63	1850.50^u^	0.51	−0.77-0.38
**Parity of women**					
**Para 0**	36(57.1%)	39(61.9%)	0.29^x^	0.59	−0.13-0.22
**Para** ≥**1**	27(42.9%)	24(38.1%)			
**Indication for cervical ripening**					
**Postdatism**	29(46%)	38(60.3%)	5.63^f^	0.23	−0.45-0.04
**Hypertensive disorders of pregnancy**	29(46%)	19(30.2%)			
**Intrauterine Growth Restriction (IUGR)**	2(3.2%)	1(1.5%)			
**Gestational Diabetes (GDM)**	3(4.8%)	3(4.8%)			
**Reduced maternal perception of fetal movement at term**	-	2(3.2%)			

Table 2 illustrates the outcome of cervical ripening and induction of labour. The mean Bishop's scores before cervical ripening in the control group (Group A) and test group (Group B) were 3.46±0.96 and 3.21±0.95, respectively.

**Table 2:** Outcome of cervical ripening/Induction of labour

	Mode of cervical ripening	Mode of cervical ripening			
	Group A; Misoprostol+Placebo (n = 63)	Group B; Misoprostol+Hyoscine (n = 63)	Test statistics	*p-value*	RR (95% CI)
**Variables**					
**Bishop's score before cervical ripening**	**3.46± 0.96**	**3.21± 0.95**	**1691** ^**u**^	**0.14**	−**0.08-0.59**
**Bishop's score after cervical ripening**	**7.59± 1.07**	**8.16± 1.51**	**1585** ^**u**^	**0.04** *	**0.35-4.12**
**Cervical ripening time (hours)**	**11.40± 7.33**	**8.48± 4.36**	**1521** ^**u**^	**0.02** *	**0.80-5.05**
**Induction** –**delivery interval (hours)**	**7.38±5.28**	**7.75±5.04**	**1761** ^**u**^	**0.54**	−**2.56-1.05**
**Oxytocin requirement**					
**Yes**	**30(47.6%)**	**25(39.7%)**	**0.81** ^**x**^	**0.37**	**1.20(0.81-1.79)**
**NO**	**33(52.4%)**	**38(60.3%)**			
					
**Mode of delivery**					
**Vaginal delivery**	**50(79.4%)**	**53(84.1%)**	**0.48** ^**x**^	**0.49**	**0.94(0.80-1.11)**
**Caesarean section**	**13(20.6%)**	**10(15.9%)**			
**Indication for Caesarean section**					
**Fetal distress**	**7(11.1%)**	**2(3.2%)**	**4.05** ^**f**^	**0.36**	**0.10-0.34**
**Cephalopelvic disproportion (CPD)**	**5(7.9%)**	**6(9.5%)**			
**Failed method of cervical ripening**	**1(1.6%)**	**1(1.6%)**			
**Maternal request**	**-**	**1(1.6%)**			
**None**	**50(79.4%)**	**53(84.1%)**			
**Complication of cervical ripening**					
**Hyperstimulation**	**2(3.2%)**	**1(1.6%)**	**5.45** ^**f**^	**0.06**	**0.33(0.11-0.98)**
**Fetal heart rate abnormality**	**10(15.9%)**	**3(4.8%)**			
**None**	**51(80.9%)**	**59(93.6%)**			
**Cervical ripening time**					
≤**12hrs**	**47(74.6%)**	**56(88.9%)**	**4.31** ^**x**^	**0.04** *	**0.01-0.28**
**>12hrs**	**16(25.4%)**	**7(11.1%)**			
**Induction -delivery interval**					
≤**12hrs**	**41(82.0%)**	**44(83.0%)**	**0.06** ^**x**^	**0.89**	−**0.15-0.12**
**>12hrs**	**9(18.0%)**	**9(17.0%)**			

* Statistically significant

u Mann Whitney u test

x Chi square

f Fisher's exact test

There was no significant difference in the two groups (p-value 0.14); however, there was a statistical difference in the post-ripening Bishop's score in the two groups [7.59± 1.07 and 8.16± 1.51 for Group A and Group B, respectively (p-value 0.04, CI 0.35-4.12)]. The mean cervical ripening time was shorter in the hyoscine group (8.48±4.36 hours) than in the placebo group (11.40±7.33 hours); p-value 0.02, CI 0.80-5.05 with 88.9% (Group B) and 74.6% (Group A) achieving a favourable Bishop's score at 12 hours (p-value 0.04, CI 0.01-0.28).

The mean induction-delivery interval in Group A was 7.38±5.28 hours, while that of Group B was 7.75±5.04 hours. This showed no statistically significant difference between the two groups (p-value 0.54), but more women in Group B (44, 83%) delivered within 12 hours of labour induction than in Group A (41, 82%); p-value 0.89, CI -0.15-0.12. More women in Group A (30, 47.6%) used oxytocin infusion than those in Group B (25, 39.7%), but this requirement for oxytocin use was not statistically significant (p-value 0.37, RR 1.20, CI 0.81-1.79).

There was no significant difference in the route of delivery, even though women in Group B (53, 84.1%) achieved more vaginal deliveries than women in Group A (50, 79.4%), p-value of 0.49. Thirteen (13) women in Group A (20.6%) had caesarean section, while 10 women (15.9%) in Group B had caesarean section, RR 0.94, CI 0.80-1.11. The indications for the caesarean sections were comparable (p-value 0.36). However, more participants in Group A (11.1%) had caesarean sections due to fetal distress as against the 3.2% in Group B.

There were recorded cases of hyperstimulation [2 (3.2%) in Group A and 1 (1.6%) in Group B] and fetal heart rate abnormality [10 (15.9%) in Group A and 3 (4.8%) in Group B]. These complications of cervical ripening and induction of labour were not statistically significant (p-value 0.06, RR 0.33, CI 0.11-0.98), but they occurred more in Group A than in Group B. One failed method of cervical ripening was reported in each of the groups (1.6%, p-value 0.36), and no uterine rupture, maternal mortality or perinatal mortality was recorded.

Table 3 shows that there were no significant differences in the neonatal outcomes, including the birth weight, Apgar score, neonatal unit (NNU) admission and indication for admission. However, more babies from the women in group A had Apgar scores less than 7 in 1 minute (22.2% as against 12.7% in group B, p-value 0.16, RR 0.59, CI -0.23-0.04) and were admitted the most in the NNU (20.6% and 9.5% for Group A and Group B respectively, p-value 0.08, RR 0.46, CI 0.19-1.14). The maternal adverse effects of the drug recorded (nausea and vomiting) were similar in both groups (p-value 1.00, RR 1.00, CI 0.49-2.05), as shown in Table 4a and Table 4b.

**Table 3:** Neonatal Outcome

	Mode of cervical ripening	Mode of cervical ripening			
	Group A; Misoprostol + Placebo (n = 63)	Group B; Misoprostol+Hyoscine (n=63)	Test statistics	*p-value*	RR (95% CI)
**Variables**					
**Birth weight (kg)**	3.25± 0.48	3.18± 0.43	0.93	0.50	−0.09-0.24
**Apgar score**					
**1min**	7.03± 1.54	7.35± 1.43	1732*	0.18	−0.84-0.21
**5mins**	8.54± 1.20	8.54± 1.54	1936*	0.76	−0.49-0.49
**Apgar score< 7**					
**1min**	14(22.2%)	8(12.7%)	1.98***	0.16	0.59(−0.23-0.04)
**5mins**	5(7.9%)	4(6.3%)	0.12**	1.00	0.80(0.23-2.84)
**Neonatal unit (NNU) admission**					
**Yes**	13(20.6%)	6(9.5%)	3.04***	0.08	0.46(0.19-1.14)
**No**	50(79.4%)	57(90.5%)			
**Indication for NNU admission**					
**Hypoglycemia**	4(6.3%)	1(1.6%)	4.08**	0.19	0.04-0.33
**Birth asphyxia**	7(11.1%)	5(7.9%)			
**Low birth weight**	2(3.2%)	-			
**None**	50(79.4%)	57(90.5%)			

**Independent sample t-test**

* Mann Whitney u test

** Fisher's exact test

*** Chi square

**Table 4:** Side effects/ adverse reactions of drug

	Mode of ripening	Mode of ripening			
	Group A; Misoprostol+Placebo (n=63)	Group B; Misoprostol+Hyoscine (n=63)	Fisher's exact test	*p-value*	95% CI
**Variables**					
**Nausea**	1(1.6%)	2(3.2%)	0.48	1.00	0.00-0.21
**Vomiting**	11(17.5%)	10(15.9%)			
**None**	51(80.9%)	51(80.9%)			

**Table 4b:** Side effects/ adverse reactions of drug

	Mode of ripening	Mode of ripening			
	Group A; Misoprostol+ Placebo (n=63)	Group B; Misoprostol Hyoscine (n=63)	Fisher's exact test	*p-value*	RR (95% CI)
**Side effect**	12(19.0%)	12(19.0%)	0.00	1.00	1.00(0.49-2.05)
**No side effect**	51(80.9%)	51(80.9%)			

## Discussion

This was a double-blind, randomized, controlled trial that assessed the effect of vaginal misoprostol plus hyoscine-n-butyl bromide versus vaginal misoprostol alone on cervical ripening time. To the best of the knowledge of the authors, this is the first randomized trial that compared the duration of cervical ripening with the use of vaginal misoprostol plus hyoscine-n-butylbromide to vaginal misoprostol alone. This inference is based on the fact that a search of the recent available scientific literature did not yield any similar previous studies for direct comparison.

The findings of this study showed that a comparison of the socio-demographic variables of the women in both groups did not show any statistically significant difference. This implies that the randomization process was effective in ensuring that the distribution of likely confounding variables was similar in both groups. Effective randomization has been demonstrated to be the most effective way to control for the effect of confounding variables in comparative studies and it is fundamental in ensuring that the findings of the study are reliable^16^.

The results of this study showed that women in the combined vaginal misoprostol and hyoscine group had a statistically significant shorter cervical ripening time than those that had vaginal misoprostol alone. This finding is in agreement with the report of Naeiji et al. 17, who conducted a randomized control trial to compare the effect of the use of Hyoscine to placebo on cervical ripening and found a statistically significant higher Bishop score in the Hyoscine group. Similarly, Tehranian et al. 18 and Ezzatalsadat et al. 19 reported statistically significant increases in the success rate of abortion and shortened duration of abortion induction, respectively, with combined intravenous Hyoscine and vaginal Misoprostol for abortion. Although their studies were in women with previable pregnancies, likely, the effect of hyoscine on both the abortion process and cervical changes during labour at term follow the same mechanism. Hyoscine-n-butylbromide is a semi-synthetic derivative of scopolamine with an anticholinergic property. It inhibits cholinergic transmissions in the abdominopelvic parasympathetic ganglia and releases spasms in the smooth muscles of the gastrointestinal and genitourinary tracts. It overcomes cervical spasm by its selective blocking action on the intramural parasympathetic ganglia of the cervico-uterine plexus, thus aids cervical dilatation without affecting uterine contractions.^9^,^17^,^20^ This mechanism of action of hyoscine explains its effectiveness on cervical ripening as obtained in this study, duration of first stage of labour^9^, abortion process^19^, and intrauterine procedures.^21^

The clinical significance of the shorter cervical ripening time obtained in this study with combined vaginal misoprostol and intramuscular hyoscine, is that this combination may be more suitable for clinical conditions where there is need to ripen the cervix faster and expedite delivery before induction of labour. This would apply to most indications for induction of labour including intrauterine fetal death, chronic medical conditions like Diabetes Mellitus and other common complications of pregnancy like prolonged pregnancy, severe pre-eclampsia, eclampsia, where delays may result in worsening of maternal or fetal conditions or both.^12^,^22^ The state of the cervix impacts on the outcome of induction of labour, thus ripening of an unfavourable cervix shortens the duration of labour and leads to a higher incidence of successful induction of labour.^7^,^12^

There was no statistically significant difference in the induction-delivery interval between the two groups in this study. This finding suggests that despite the statistically significantly higher mean Bishop's score in the Misoprostol plus hyoscine group, this did not affect the induction delivery interval. This implies that it is more important for the Bishop's score to be in the favourable range, and once this is achieved, the individual progress of women in labour does not differ significantly. This is similar to the findings of Fox et al.^23^ They conducted a meta-analysis involving nine studies on the use of intravaginal misoprostol and Foley's catheter on cervical ripening and induction of labour and reported that despite having a statistically significant higher mean Bishop's score, there was no corresponding significant difference in the induction-delivery interval because all the women in both groups had achieved a favourable Bishop's score before commencement of induction.^23^ This was also corroborated by the findings of Burodo et al^24^ in a prospective cross-sectional study on the outcome of induction of labour in a tertiary hospital in North Western Nigeria.

This study demonstrated no significant difference between the two groups in terms of rate of vaginal delivery and rate of operative delivery/caesarean section and its indications. This was similar to the findings of Hanna et al^8^, Ejikeme et al^9^, Naeiji et al^17^, and Ahmed et al.^25^ This study also reported no significant difference between the two groups as regards adverse maternal and fetal outcomes, which is in keeping with other previous studies.^9^, ^17^, ^25^ The clinical implication of this, is that hyoscine only has a significant effect in achieving cervical ripening with no significant effect on the outcome of the labour once its administration has been stopped, and it is safe to both parturient and foetus. This conforms to the primary aim of cervical ripening and induction of labour, which is to achieve safe vaginal delivery.^26^ The effect of hyoscine in reducing the cervical ripening time without maternal or perinatal morbidity and mortality, as recorded in this study, could result in reduced periods of anxiety, shorter hospital stays, reduced hospital cost and ultimately fulfilled childbirth experience. Hyoscine-n-butyl bromide is cheap and readily available in our environment. Any cervical ripening method or an adjunct that can prepare the cervix for timely, successful delivery in a safe, effective, inexpensive and feasible manner may be valuable, especially in the context of providing patient-centred care to increase family convenience and satisfaction, and decreasing healthcare costs and resource allocations, which is a priority for health systems worldwide.^17^

The strength of this study lies in the fact that it was a double-blind, randomized controlled trial, hence minimising the possible effects of confounders and bias on the findings of the study. However, there were some challenges as regards knowing the exact time of transition of the cervix from an unfavourable to a favourable status, the use of a divided 200µg misoprostol tablet instead of a single 25µg misoprostol tablet and the possibility of intra-and inter-observer variation in the assessment of Bishop's score. These limitations were minimized by the use of scored misoprostol tablet and the pharmacist cutting it into almost exactly equal parts and for the fact that the drugs were administered to all the women without knowing the arm of the study they belonged to. The intra- and inter-observer variation in the assessment of the Bishop's score was minimized by training and re-training of research assistants on the use of a standard protocol for the assessment of the different outcome measures.^27^

## Conclusion

This study found that combined 20mg intramuscular Hyoscine and 25µg vaginal Misoprostol was effective in reducing cervical ripening time when compared to the use of 25µg vaginal Misoprostol alone, without any significant adverse maternal or neonatal outcome.
